# Cell-Surface Inter-Cytochrome Electron Transfer Limits Biofilm Electron Conduction Kinetics in *Shewanella oneidensis*

**DOI:** 10.1021/jacs.5c10357

**Published:** 2025-12-15

**Authors:** Xinxin Wen, Xizi Long, Wenyuan Huang, Masahiro Kuramochi, Akihiro Okamoto

**Affiliations:** † Research Center for Macromolecules and Biomaterials, 52747 National Institute for Materials Science, Tsukuba, Ibaraki 305-0044, Japan; ‡ Key Laboratory of Typical Environmental Pollution and Health Hazards of Hunan Province, School of Public Health, Hengyang Medical School, University of South China, Hengyang 421001, China; § Graduate School of Chemical Sciences and Engineering, Hokkaido University, North 13 West 8, Kitaku, Sapporo, Hokkaido 060-8628, Japan; ∥ Graduate School of Science and Engineering, Ibaraki University, Hitachi, Ibaraki 316-8511, Japan; ⊥ Graduate School of Frontier Sciences, 105219 The University of Tokyo, Kashiwa, Chiba 277-8561, Japan; # Graduate School of Science and Technology, University of Tsukuba, 1-1-1 Tennodai, Tsukuba 305-8577, Japan; ¶ Research Center for Autonomous Systems Materialogy, Institute of Integrated Research, Institute of Science Tokyo (Science Tokyo), 4259 Nagatsuta-cho, Midori-ku, Yokohama, Kanagawa 226-8503, Japan

## Abstract

The electrical conductivity of biofilms plays a critical role in advancing bioelectronics for energy and environmental applications, yet the underlying mechanisms remain poorly understood. Previous studies proposed interheme electron transfer between hemes 5 and 10 in the outer-membrane deca-heme cytochrome (OMC) MtrC as the rate-limiting step in the biofilm electron conduction of *Shewanella oneidensis* MR-1. However, the strong interheme electron coupling in MtrC suggests that interprotein interactions may represent the primary barrier to biofilm electron conduction. Here, we investigated the biofilm electron conduction mechanism with a focus on interprotein electron transfer in *S. oneidensis* MR-1. Conductive currents and their temperature dependence were measured for estimating the thermal activation energy (*E*_a_) by using indium tin-doped oxide (ITO) interdigitated electrodes in wild-type and mutant biofilms. While deletion of periplasmic cytochromes had a negligible impact on *E*_a_, the deletion of OmcA or MtrC increased *E*_a_ 3-fold, revealing that interprotein interactions, particularly between OmcA and MtrC, dominate biofilm electron transfer over clonal OMC interactions. Furthermore, suppressing outer-membrane fluidity dramatically increased *E*_a_, while interheme exciton coupling negligibly changed in the OMCs, confirming the critical role of protein diffusion and collision on the outer membrane. Flavin binding to OmcA or MtrC reduced conduction currents attributable to heme centers but enhanced those assignable to noncovalently bound flavins, suggesting that flavin occupancy blocks hemes 2 and 7, which serve as key interprotein electron transfer sites. These findings provide a mechanistic foundation for engineering highly conductive biofilms through targeted protein interface optimization, offering new avenues for the development of bioelectronic technologies.

## Graphical abstract

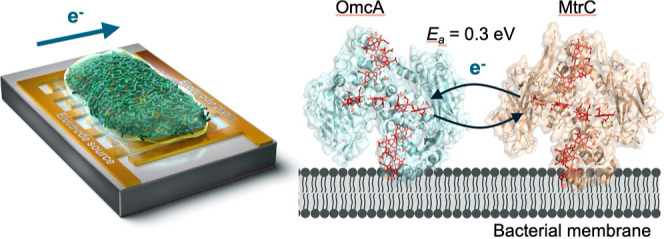

## Introduction

Electrochemically active bacteria, with the ability to form electrically conductive biofilm matrices for bioenergy conversion and bioelectronics,^1^ have engendered much interest in the scientific community; therefore, understanding the flow of electrons inside the biofilm is crucial to achieving high current density in microbial electrochemical systems.^2^ Significant progress has been made in elucidating the molecular structures of redox-active proteins within the extracellular matrix,^3,4^ yet the kinetic bottlenecks in long-distance electron transfer (LDET) across the densely packed, multilayered biofilms remain poorly understood.^5–40^ Among electrogenic organisms,*Shewanella oneidensis* MR-1 (*S*.MR-1) stands out, known for facilitating robust extracellular electron transfer (EET) to both electrodes and minerals via its intricate outer membrane cytochrome complexes (OMCs), including MtrC and OmcA. These complexes, which function akin to biological wires, are equipped with extensive networks of heme reaction centers that efficiently channel electrons from the cellular interior to the exterior.^6^ Previous studies have inferred the role of OMCs in mediating LDET within biofilms through detailed analyses of redox signal.^7^ The unique configuration of heme centers along the surface of MtrC and OmcA enables multiple electron ingress and egress points (Figure 1A),^6,32^ thereby facilitating the formation of expansive electron transport routes through diffusion and intermolecular collisions on the membrane’s surface.^8,9^ However, defining the rate-limiting steps in electron transfer, whether intra- or inter-OMCs, continues to challenge researchers. Recent work by Xu et al. highlighted the potential rate-limiting pathways by employing an interdigitated electrode system to ascertain the temperature dependence of biofilm conductivity, revealing insights into the activation energy (*E*_a_) associated with the rate-limiting step of the biofilm electron conduction.^10,11^ Their analysis suggests that the electron routes in the hemes 5–10 pathway of MtrC, characterized by *E*_a_ values comparable to the kinetic Monte Carlo model simulation and higher than those of alternate routes, are likely critical. This approach, however, did not explore other potential electron transfer pathways involving periplasmic cytochromes or inter-OMCs interactions comprehensively (Figure 1B). Our investigation builds on this foundation by contrasting gene knockout mutant strains to examine the impact of intercytochrome interactions on the rate-limiting step energy barrier, *E*_a_, for biofilm electron dynamics, rather than conduction current (*I*_cond_), which could vary with other factors such as biofilm thickness.^7^ We also probe the effects of OMCs diffusion and collision dynamics with cholesterol^12^ and the interaction of noncovalently bound flavin with OMCs on the biofilm electron conduction, associated with whole-cell circular dichroism (CD) spectroscopy, to explore interheme exciton coupling in OMCs.^13^ Pinpointing and clarifying these bottlenecks will reveal strategies that significantly enhance electron transfer within biofilms, thereby augmenting the operational efficacy of bioelectronic devices.

**Figure 1:**
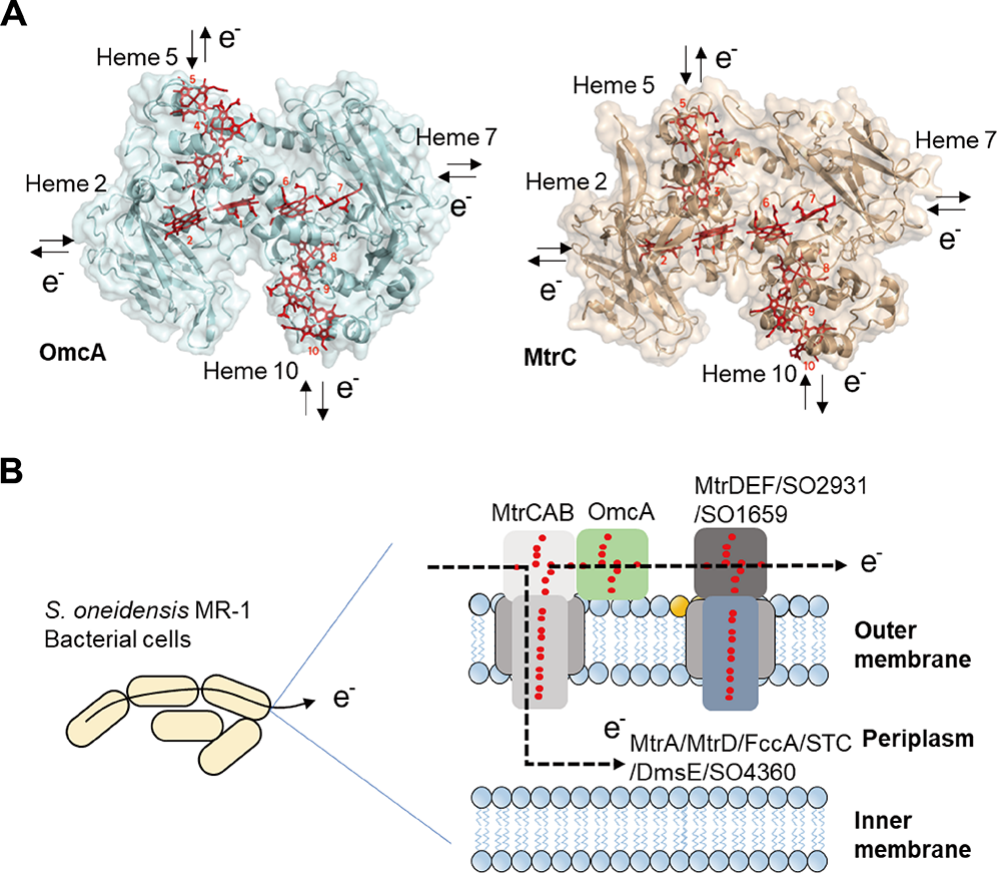
(A) Crystal structure of OmcA (PDB: 4LMH) and MtrC (PDB: 4LM8). Heme centers for electron ingress and egress points are highlighted. (B) Schematic model for potential intercellular electron pathways through the outer membrane or the periplasmic cytochromes.

## Experimental Methods

### Microbe Culture Conditions

*S.*MR-1 wild type (WT) and mutant strains were inoculated from our lab storage and precultured in a 50 mL falcon tube with Luria–Bertani (LB) medium (shaking at 180 rpm, 30 °C). Then, the cell pellets were washed two times with a defined medium (DM). Cells were harvested after being washed two times with DM. The compositions of DM are NaHCO_3_ 2.5 g/L, CaCl_2_·2H_2_O 0.08 g/L, NH_4_Cl 1.0 g/L, MgCl_2_·6H_2_O 0.2 g/L, NaCl 10 g/L, and HEPES 7.2 g/L. The methods to construct Δ*omcAll* (lacking the genes encoding OMC: *mtrB, mtrC, mtrD, mtrE, mtrF, and omcA*), Δ*PEC* (lacking the genes encoding the periplasmic cytochrome genes: *mtrA, mtrD, dmsE*, SO4360, and *cctA*), Δ*mtrC*/Δ*omcA*, Δ*omcA,* and Δ*mtrC* were described in previous studies.^14^ In addition, *mtrC* was reintroduced into the Δ*mtrC* and Δ*mtrC*/Δ*omcA* using plasmid pETSXM2-pLacI-MtrC (Plasmid #174615, Addgene) to construct strains Δ*mtrC*(pMtrC) and Δ*mtrC*/Δ*omcA*(pMtrC), respectively.

### Electrochemistry Measurements

A single-chamber cell was used for *I*_cond_ measurement, similarly to the previous study.^11^ The interdigitated electrode (IDE) of indium tin-doped oxide (ITO) was sputter deposited on a glass with a 10 μm gap between the source and drain electrodes, WE1 and WE2, respectively (10 μm in width) (Figure 2A and S1). The reference was an Ag/AgCl electrode (saturated KCl), and the counter electrode was a Pt wire. The 6 mL washed cells were dispersed in DM to OD_600_ 1.0 and then they were purged with nitrogen for 30 min to remove the oxygen. The cell suspensions were electrochemically incubated for 24 h at 0.4 V (vs standard hydrogen electrode [SHE]) to form the biofilm on an IDE electrode (VMP3: Biologic SAS, Seyssinet-Pariset, France). Then, *I*_cond_ was obtained by setting the source and drain potentials, *E*_S_ and *E*_D_, respectively, in a constant offset voltage of 20 mV. The current was subtracted by the equation below^11^

1 $$I_{cond}=\left(I_{drain}-I_{source}\right)/2$$

*I*_drain_ and *I*_source_ are the current observed during sweeping electrode potential range from −300 mV to 400 mV with higher and lower potential, respectively.

**Figure 2:**
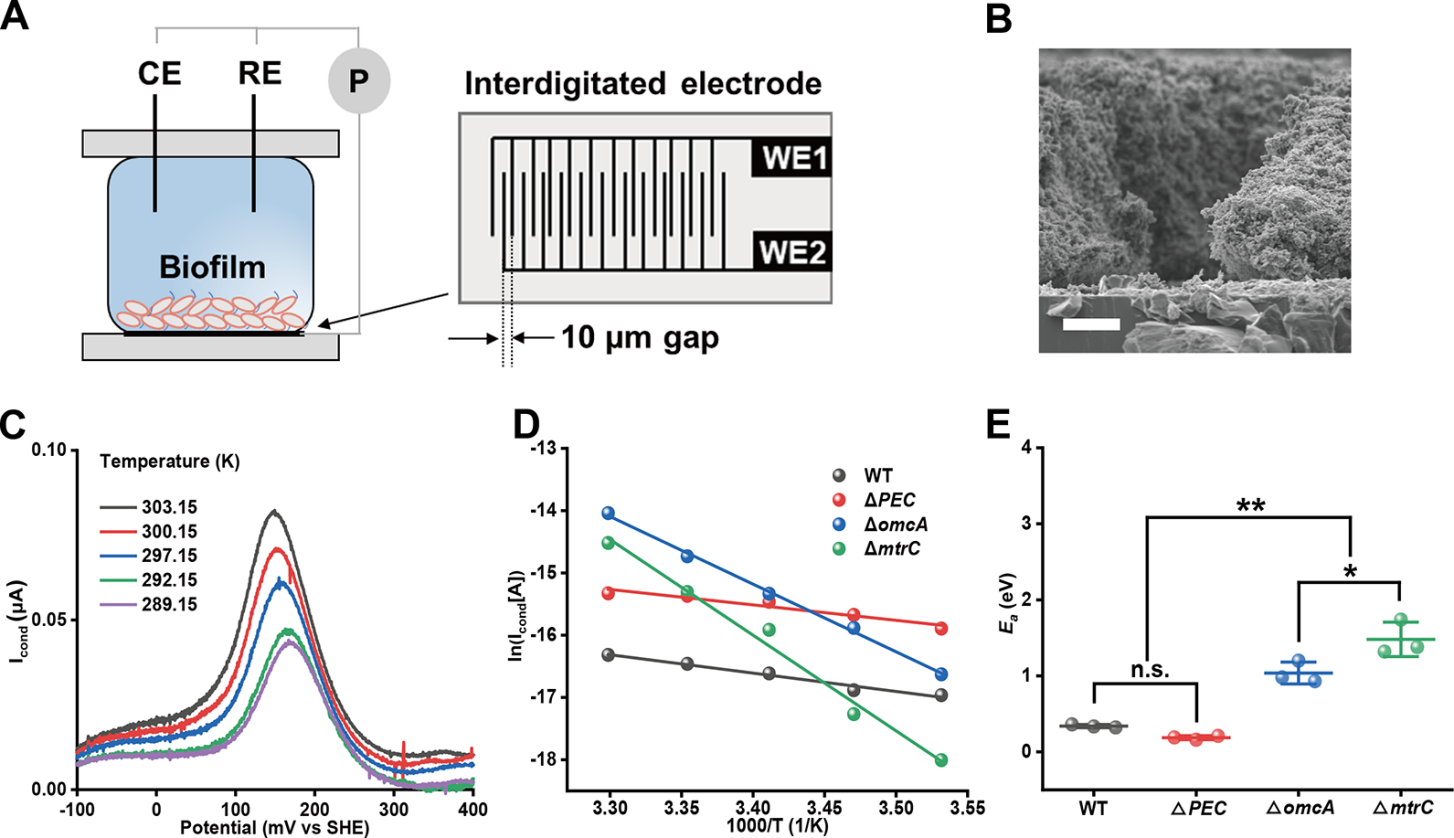
Conductive current measurements for the biofilm of *Shewanella oneidensis* MR-1 (*S.*MR-1). (A) *S.*MR-1 cells were electrically cultured for 24 h to form the biofilm bridging the indium tin-doped oxide (ITO) interdigitated electrodes with 10 μm gaps. (B) Scanning electron microscope images of biofilm grown and remaining on the ITO electrodes after washing processes. The white bar is ten μm. (C) Representative conduction current (*I*_cond_) versus the gate potential under each temperature, calculated by subtracting the source from the drain current in the absence of an electron donor (offset voltage = 20 mV, scan rate ν = 1 mV s^–1^). The same tendency was observed in three independent assays. (D) Representative Arrhenius-style plots of wild type (WT), Δ*PEC*, Δ*omcA*, and Δ*mtrC*. (E) The semiempirical thermal activation energies (*E*_a_) of WT, Δ*PEC*, Δ*omcA* and Δ*mtrC*. The *E*_a_ was calculated according to eq 2. Data in panel E are presented as means ± standard deviation (s.d.) (*n* = 3). n.s., not significant, *, *p* < 0.05, and **, *p* < 0.01.

The *E*_a_ accounting for the redox-mediated electron transport of *S.*MR-1 WT and mutant strains were acquired by conducting temperature-linked Arrhenius equation examinations. Temperatures were stepwise controlled from 30 to 9 °C by circulating water in the jacket of the single-chamber reactor. After stabilizing temperature for 15 to 20 min, the gate measurements were conducted. The *E*_a_ was obtained by fitting the data to the empirical Arrhenius equation

2 $$\ln\left(I_{cond}\right)=A-1000/T\times \left(E_{a}/1000k\right)$$

where *A* is the pre-exponential factor, *T* is the temperature (K), and *k* is the Boltzmann constant.

### Scanning Electron Microscopy (SEM)

The biofilm incubated on the electrode was fixed with 2.5% glutaraldehyde for 5 days at 4 °C. Then, samples were dehydrated in 25, 50, 75, 90, and 100% ethanol and freeze-dried with t-butanol under a vacuum before SEM. The dried samples were coated with evaporated platinum for 30 s and were then checked by a Keyence VE-9800 microscope at 10 kV.

### Cellular Cholesterol Quantification

*S.*MR-1 cells were treated with 0, 20, 100, or 200 mg/L cholesterol and incubated in an electrochemical reactor at 0.4 V and 30 °C for 20 h. Cells were harvested by centrifugation at 6,000 rpm (3,830*g*) for 5 min at 4 °C both before and after the electrochemical assay. The resulting pellets were lysed by resuspension in cold ethanol: isopropanol (1:1, v/v), followed by vortexing for 30 s and incubation at −20 °C for 1 h. Lysates were centrifuged at 12,000 rpm (15,000*g*) for 10 min, and supernatants were collected. After vacuum drying, the residues were resuspended in 1 mL of Assay Buffer II and used for subsequent analysis. Cellular cholesterol levels were measured using the Total Cholesterol & Cholesteryl Ester Colorimetric Assay Kit (ab282928, Abcam, Cambridge, UK) according to the manufacturer’s instructions.

### Fluorescence Recovery after Photobleaching (FRAP)

*S.*MR-1 cells were treated with 0, 20, 100, or 200 mg/L cholesterol and incubated in an electrochemical reactor at 0.4 V and 30 °C for 20 h. After incubation, the entire culture was harvested and resuspended in 1× phosphate-buffered saline (PBS) to OD_600_ 1.0. The outer membrane was stained by adding FM4-64FX dye to a final concentration of 30 μg/mL, followed by incubation in the dark for 20 min. A 30 μL aliquot of the stained suspension was placed on a glass slide for imaging. Fluorescence imaging was performed using a Nikon A1 confocal microscope equipped with a 60× objective lens. The dye was visualized using 488 nm excitation light and an emission filter with a bandwidth of 663–738 nm. A defined region of interest (ROI) on the outer membrane was photobleached using a 488 nm laser at full power for 0.5 s. Fluorescence recovery within the ROI was recorded over 80 s postbleaching. Fluorescence intensity in the bleached area was normalized to that of an adjacent unbleached control region. Recovery kinetics were fitted to a single-exponential function to determine the characteristic recovery time constant (τ), as described previously^15^

3 $$F\left(t\right)=A\times \left(1-e^{-t/\tau }\right)$$

### Circular Dichroism (CD) Spectroscopy

The CD spectra of the OMCs in an intact cell were measured by the Jasco spectropolarimeter (JASCO CD, J-1500: Tokyo, Japan). Briefly, the parameters were set as 200 nm min^–1^, 0.1 nm data pitch, and 5.0 nm bandwidth. Cell suspensions of *S.*MR-1 WT and mutant strains Δ*omcAll*, Δ*omcA*, and Δ*mtrC* were prepared by washing and resuspending in DM. To further enhance the signal, we increased the cell density to OD_600_ 1.32 and used an integrating sphere (IS) to collect the scattering light from the cell suspension in front of the CD detector. Three mL of cell suspension was added into a quartz cuvette and was purged with nitrogen for 10 min after adding 30 mM sodium lactate as an electron donor to reduce the OMCs. In contrast, to oxidize heme centers in OMCs, the cell suspension was incubated in the presence of oxygen and fumarate (50 mM). The temperature of the suspensions was set at 36 °C, 31 °C, 26 °C, 21 °C, 16 °C, and 11 °C by the accessory equipped with the Peltier-type temperature controller (PTC-517: JASCO, Tokyo, Japan). Upon reaching the setting temperature, the scanning of the suspensions was conducted. Then, their ellipticities were normalized at 440 nm.^16^

## Results

### OMCs Mediate Biofilm Electron Conduction

To examine the contribution of the outer membrane or the periplasmic cytochromes on LDET inside the biofilm of *S.*MR-1, we first compared biofilm conduction currents among WT, Δ*omcAll* and Δ*PEC* by bipotentially controlling the *E*_S_ and *E*_D_. A single-chamber four-electrode system with an ITO interdigitated electrode with 10 μm gap was used to monitor the *I*_cond_ from the *S.*MR-1 biofilm (Figures 2A and S1). Following incubation at 0.4 V (vs SHE) at 30 °C, biofilm formation with a thickness of several tens of micrometers on the electrode was observed, as reported (Figure 2B).^7^ We washed the electrode surface twice to diminish the interference of metabolic current toward *I*_cond_, resulting in low current production (Figure S2). We then measured *I*_drain_ and *I*_source_ by sweeping the voltammetry to calculate *I*_cond_ by eq 1. As shown in Figure 2C, the oxidative peak of the gate potential *E*_G_ (*E*_G_ = [*E*_S_ + *E*_D_]/2) at around 10 mV from the *I*_cond_ of *S.*MR-1 WT was observed. Upon altering the reactor temperature from 30 to 9 °C, we confirmed the linear correlation between ln (*I*_cond_) and 1000/T (Figure 2D). The magnitude of the energy barrier derived from the Arrhenius equation was estimated, as previously reported (eq 2), resulting in the semiempirical thermal *E*_a_ of 0.34 ± 0.02 eV (Figure 2E), consistent with electron transfer reactions involving hemes of *S.*MR-1 OMCs.^11^

The *I*_cond_ profile and temperature dependency were distinct in Δ*omcAll* but similar in Δ*PEC* compared with WT (Figures 2D, S3 and S4). *I*_cond_ of the Δ*omcAll* strain was disordered, lacking the clear Arrhenius behavior observed in WT upon shifting the reactor temperature (Figure S3), which makes it infeasible to derive a reliable *E*_a_. In contrast, ΔPEC showed almost identical peak positions and *E*_a_ values with WT (Figures 2D,E and S4). These results suggest that OMCs rather than the cytochromes in the periplasm mediate the biofilm conduction current in WT.

### The OmcA and MtrC Interaction Facilitates the Biofilm Electron Conduction

Given the expression of MtrDEF is limited compared with OMCs in WT,^17^ the observed *I*_cond_ is most likely attributable to OmcA and MtrC on the cell surface (Figure 1). To elaborate on the interprotein electron transport via OMCs, we measured the *I*_cond_ of strains Δ*omcA* and Δ*mtrC* biofilm. At 30 °C, the *I*_cond_ was comparable to that of the WT (Figure S5). However, at reduced temperatures, the *I*_cond_ values of Δ*omcA* and Δ*mtrC* biofilm decreased sharply (Figure 2D), corresponding to an increase in the *E*_a_ to 1.04 ± 0.14 eV (Δ*omcA*) and 1.48 ± 0.23 eV (Δ*mtrC*), respectively (Figures 2E and S5). The Δ*mtrC*/Δ*omcA* double mutant did not exhibit Arrhenius-type behavior (Figure S6A). These results suggest that at least one of the proteins, MtrC or OmcA, is essential for biofilm electron transfer, while the presence of both enhances this process. Accordingly, both complemented strains (Δ*mtrC* (pMtrC) and Δ*mtrC/*Δ*omcA* (pMtrC)) exhibited temperature-dependent conduction consistent with the Arrhenius relationship (Figure S7A–C). The Δ*mtrC* (pMtrC) strain had an *E*_a_ of 1.00 ± 0.01 eV, while Δ*mtrC/*Δ*omcA* (pMtrC) had a higher *E*_a_ of 1.38 ± 0.07 eV (Figure S7D), suggesting that absence of OmcA (Figure S7E) raised the thermodynamic barrier for electron transfer kinetics in the biofilm. The higher *E*_a_ in the Δ*mtrC* (pMtrC) strain than WT likely results from differences in protein localization^4^ and surface exposure due to the plasmid-driven expression of MtrC.^41^ Given these mutations of OMCs do not impact heme alignment in MtrC and OmcA,^13^ significantly higher *E*_a_ of Δ*omcA* and Δ*mtrC* than WT is likely due to the loss of protein–protein interactions between OmcA and MtrC in the mutant strains. Therefore, electron transport between identical proteins (OmcA–OmcA or MtrC–MtrC) requires a higher *E*_a_ than OmcA-MtrC electron transfer. This critical effect of OmcA or MtrC deletion implies the negligible impact of MtrDEF as well. Notably, Δ*omcA* and Δ*mtrC* showed a slight negative shift, ∼20 mV, in the *I*_cond_ peak potential, but the midpoint potential observed in cyclic voltammetry shifted ∼80 mV (Figure S8), further supporting that the rate-limiting step for *I*_cond_ is interprotein electron transfer but not the OMCs-electrode interfacial electron transfer. Additionally, the near-identical concentration of heme centers on the cell surface quantified by cyclic voltammetry (Figure S8) further supports our idea, as a concentration-dependent effect such as mechanical pressure can be neglected in the mutant strains.^16^

### Contribution of Diffusion and Collision Among OMCs on the Biofilm Electron Conduction

To confirm that the interprotein interaction via the diffusion and collision of OMCs dictates the *E*_a_, we decreased membrane fluidity with cholesterol that specifically interacts with lipid membrane (Figure 3A).^12^ Cholesterol incorporation into *S.*MR-1 cells was confirmed by quantifying cellular cholesterol levels using a standard calibration curve (Figure S9A), following cell lysis and analysis with a cholesterol assay kit after cholesterol preculturing. A dose-dependent increase in intracellular cholesterol was observed, with significant accumulation at concentrations of 100 and 200 mg/L compared to untreated controls (Figure 3B), indicating the successful integration of cholesterol into the membrane.

**Figure 3:**
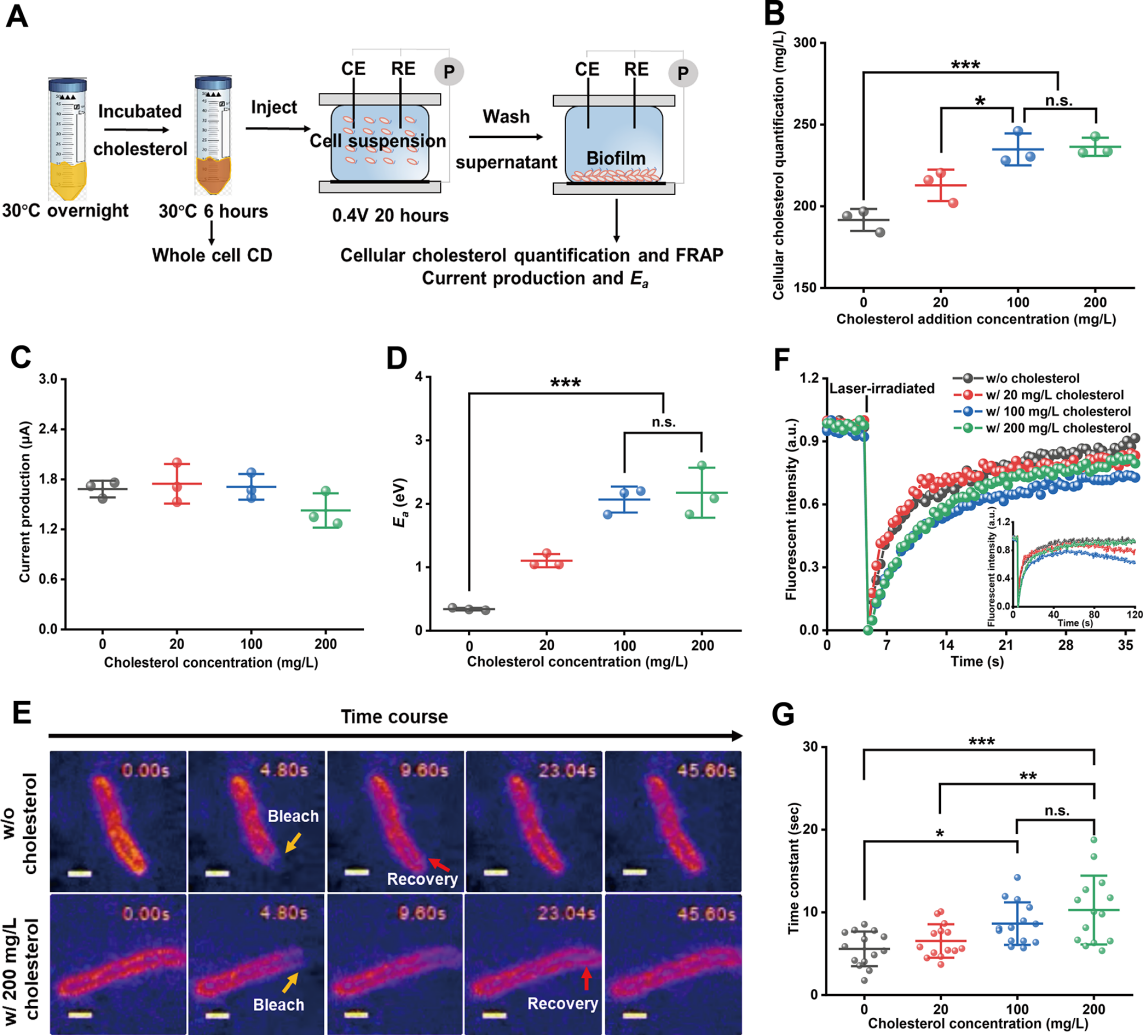
Cholesterol modulates outer membrane fluidity and augments *E*_a_ for *I*_cond_ in *S*.MR-1 biofilm. (A) Scheme steps of electrochemical assays after preculturing with cholesterol. (B) Quantification of intracellular cholesterol levels using a standard calibration curve before electrochemical incubation. (C) Current production of the WT under different cholesterol concentrations. The maximum current was selected for comparison (*n* = 3). Data are presented as mean ± s.d. (D) *E*_a_ in the absence and presence of cholesterol is presented as mean ± s.d. (*n* = 3). n.s., not significant, ***p < 0.001. (E) Representative Time-lapse Fluorescence Recovery After Photobleaching (FRAP) images of FM4-64FX-labeled S.MR-1 cells at cholesterol concentrations of 0 and 200 mg/L. The scale bar is one μm. (F) FRAP images illustrating outer membrane fluorescence recovery at cholesterol concentrations of 0, 20, 100, and 200 mg/L. (G) Quantification of fluorescence recovery time and diffusion time constants (τ) at a varying cholesterol concentration. Data are presented as mean ± s.d. (*n* = 14). n.s., not significant, *p < 0.05, ** *p* < 0.01, and *** *p* < 0.001.

Notably, preincubation with cholesterol had a negligible effect on the microbial current production from lactate oxidation in *S*.MR-1 cells (Figures 3C and S10), indicating that the electron transfer capability to the electrode via the OMCs was unaffected. However, the estimated *E*_a_ markedly increased following the cholesterol treatment. Specifically, *E*_a_ increased from 0.34 ± 0.03 eV in the control group to 1.10 ± 0.10 eV at 20 mg/L cholesterol and further to approximately 2.07 ± 0.21 eV at 100 mg/L cholesterol (Figure 3D). No significant difference in *E*_a_ was observed between the 100 and 200 mg/L (2.17 ± 0.39 eV) cholesterol treatments (Figure 3D). Consistently, this tendency aligned with the intracellular cholesterol level after electrochemical treatment (Figure S9B). Such a significant increase in *E*_a_ is unlikely to be attributable to reduced lipid diffusion, as the *E*_a_ for lipid diffusion typically falls within 2 orders of magnitude lower.^18^ Additionally, cholesterol addition did not produce any signals in the absence of OmcA and MtrC (Δ*mtrC/*Δ*omcA*) (Figure S6B), indicating that cholesterol does not affect the assignment of the OmcA-MtrC signal. These findings strongly suggest that the lateral diffusion and transient collision among OMCs play a critical role in mediating efficient interprotein electron transfer within the biofilm matrix.

To confirm the impact of reduced membrane fluidity on protein dynamics, FRAP was performed (Figures 3E,F and S11). In cholesterol-free cells, full fluorescence recovery occurred within 9.6 s, whereas cells treated with 200 mg/L cholesterol exhibited a significantly slower recovery, reaching completion at approximately 23.0 s (Figure 3E). Similarly, intermediate cholesterol concentrations (20 and 100 mg/L) also delayed recovery relative to the control (Figure S11). Quantitative analysis showed that the diffusion time constant (τ) increased progressively with the cholesterol concentration (Figure 3G). Statistical analysis revealed differences between the 0 mg/L and 100 mg/L groups (*p* < 0.05), as well as between the 0 mg/L and 200 mg/L groups (*p* < 0.001); however, no significant difference was found between the 100 mg/L and 200 mg/L groups. These results indicate that cholesterol incorporation reduced the lateral mobility of membrane proteins.

Additionally, we validated that the cholesterol does not impact the interheme coupling in OMCs with the CD spectroscopy.^19^ Given that the stacked ten ferrous porphyrins inside the OMCs have 2 orders of magnitude higher Soret absorption of CD spectra than monoheme horse heart cytochrome based on the strong exciton coupling effect from the heme coupling, the variation of CD signal reflects the tiny changes of interheme distance and spatial orientation.^13^ Strong Soret absorption (at ∼400–550 nm) observed in CD spectra of WT and OMCs mutants remained unchanged upon incubation in the presence of cholesterol (Figure S12). These results indicate that cholesterol did not affect the interheme coupling within the OMCs, strongly supporting our idea that *E*_a_ relies on the interprotein electron transfer via diffusion and collision among the OMCs.

### Flavin Binding Reveals the Electron Transfer Pathway in OMCs

To investigate the heme responsible for the interfacial electron transfer in OMCs in biofilm electron conduction, we examined the impact of flavin binding to the OMCs. The staggered-cross structure of MtrC and OmcA suggests that four terminal hemes (2, 5, 7, and 10) serve as potential ingress/egress sites (Figure 1A). Among these, in vitro spectroscopy, crystallography, and modeling studies demonstrated that hemes 2 and 7 were regarded as the reactive points, being more buried under the barrel motif, selectively bound with noncovalently bound cofactors such as flavins.^6,20–22^ We hypothesized that flavin binding would form a flavin-heme complex which blocks these hemes, reducing electron transfer through them while leaving hemes 5 and 10 open.

Upon adding two μM riboflavin (RF) or flavin mononucleotide (FMN) to *S.*MR-1 biofilms on interdigitated electrodes, we observed an immediate decrease in the *I*_cond_ peak associated with heme-mediated conduction (Figure 4A,B). This rapid response aligns with the known flavin-OMC binding kinetics (*K*_d_ ∼ 10 μM)^23^ but is inconsistent with slower processes such as gene expression changes. Although RF and FMN have similar affinities for OMCs, FMN elicited a greater reduction in *I*_cond_ at the same concentration, suggesting that FMN may more effectively alter inter-OMC interactions and inhibit interheme electron transfer.

**Figure 4:**
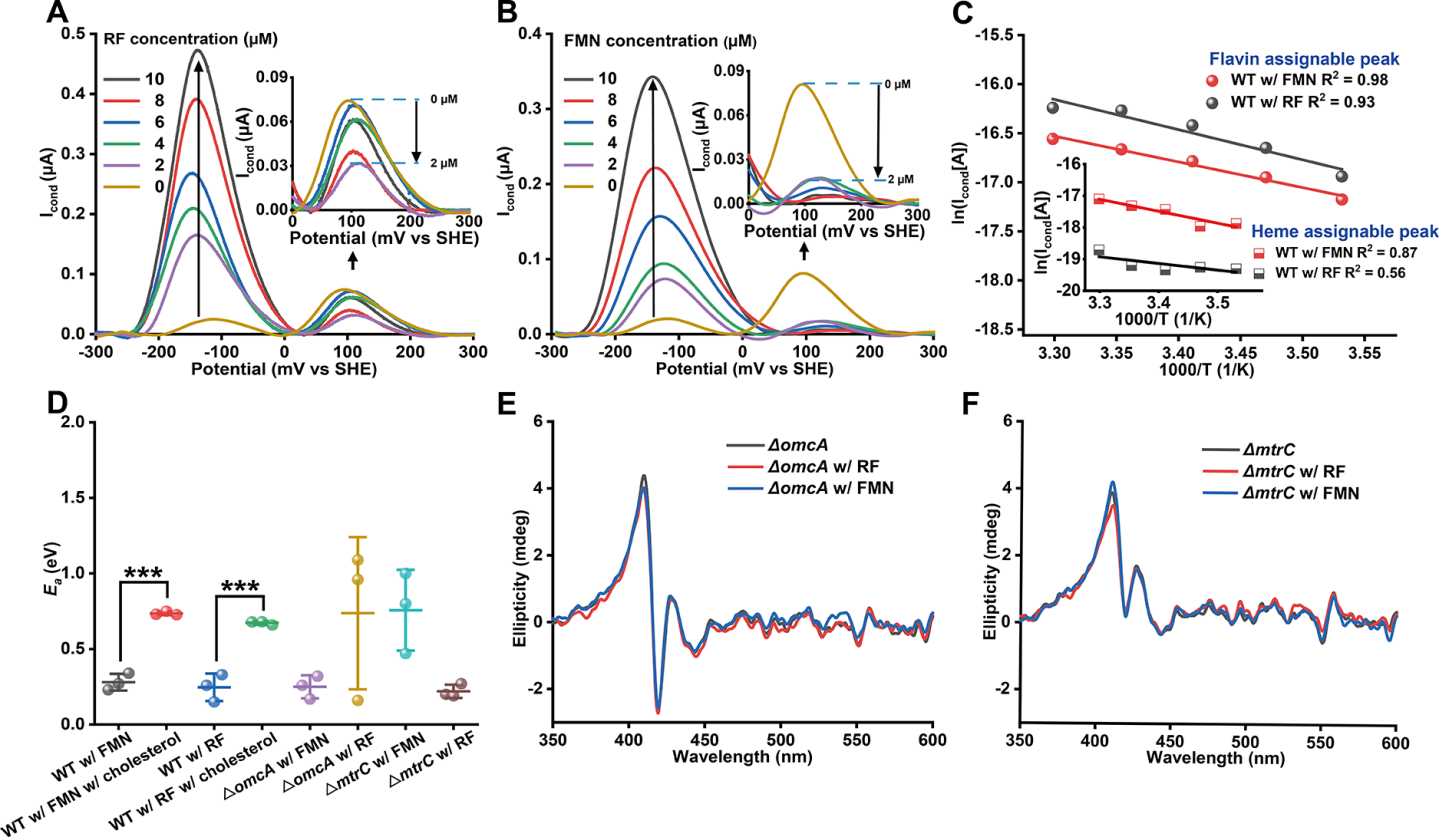
Effect of flavin addition on the biofilm electric conduction. *I*_cond_ of *S.*MR-1 biofilm versus the gate potential with increasing concentrations of riboflavin (RF) (A) and flavin mononucleotide (FMN) (B). (C) Arrhenius-style plots of *I*_cond_ assignable for heme and flavin peaks in the presence of 10 μM RF and FMN. (D) *E*_a_ and its standard error means (*n* = 3) for *I*_cond_ assignable to flavin peaks with WT and mutant strains, Δ*omcA* and Δ*mtrC*, in the presence of 10 μM FMN and RF. Cholesterol (200 mg/L) was added for WT with 10 μM FMN or RF condition. ***p < 0.001. (E,F) Circular dichroism spectra of *S.*MR-1 mutant strains Δ*omcA* and Δ*mtrC* in the presence and absence of 10 μ MRF or FMN.

Increasing the flavin concentration beyond 2 μM did not result in proportional decreases in *I*_cond_. This may reflect the complexity of flavin-mediated disruption of cytochrome interactions, and the prolonged time course of the flavin titration (60 min per addition), during which the expression of OMCs may shift.^17^ Notably, the temperature dependence of the *I*_cond_ peak was also disrupted in the presence of flavins. Unlike the linear relationship observed under flavin-free conditions, heme-mediated conduction deviated from Arrhenius behavior (Figures 4C and S13), consistent with increased heterogeneity or dynamic instability in interprotein electron transfer. Given that noncovalently bound flavins were confirmed under identical experimental conditions,^24,25^ these results strongly suggest that binding of flavins to OMCs diminishes the contribution of hemes to conduction.

In contrast, a distinct electron transfer profile was observed upon flavin addition, as evidenced by a new *I*_cond_ peak at −150 mV that increased in a concentration-dependent manner with either RF or FMN (Figure 4A,B). This potential closely matches the redox potential of noncovalently bound flavins, implicating a semiquinone-mediated one-electron transfer process.^24^ Supporting this, cell-free flavin solutions exhibited multiple *I*_cond_ peaks (Figure S14). Additionally, treatment with 50 μM carbonyl cyanide *m*-chlorophenylhydrazone, an antibiotic agent, abolished this peak (Figure S15), consistent with that RF binding to OMCs is stabilized by active microbial respiration^25,26^ and reduced heme center.^21,24^ These findings confirm that the −150 mV peak arises predominantly from bound flavins rather than from free flavins diffusing nonspecifically through the biofilm matrix.

This flavin-mediated pathway exhibited a linear temperature dependence with an *E*_a_ value of ∼0.3 eV, which is slightly lower than that observed for WT biofilms in the absence of flavins. In contrast, no such correlation was observed in cell-free flavin solutions (Figure S14), reinforcing that the conductive behavior at −150 mV is specific to flavin-OMC interactions rather than nonspecific redox mediation. The emergence of this distinct conduction mechanism further substantiates the formation of noncovalently bound flavins in our system, suggesting that the marked impact of flavin addition on heme-mediated *I*_cond_ is caused by blocking hemes 2 and 7 in MtrC and OmcA.

### Low Energy Barrier for Biofilm Conduction Among Monoclonal OMCs via Noncovalently Bound Flavins

While the FMN and RF specifically bind to MtrC and OmcA, respectively, at the microbe-ITO electrode interface,^23^ these affinities may vary under different electrochemical conditions.^27^ To examine the binding specificity of flavins under biofilm conditions, we evaluated their effects using Δ*omcA* and Δ*mtrC* mutant biofilms.

Despite the previously reported specificity, both RF and FMN similarly suppressed heme-mediated *I*_cond_ near +100 mV in both Δ*mtrC* and Δ*omcA* mutants (Figures S16 and S17), suggesting that flavins nonselectively associate with either OMC to block direct heme–heme electron flow. Consistently, both mutants exhibited a −150 mV *I*_cond_ peak upon addition of either RF or FMN. These findings indicate that the flavin-binding preference observed on electrode surfaces does not strictly hold within the biofilm context and further imply that the −150 mV *I*_cond_ is not limited by the electron transfer at the microbe-electrode interface but rather by interprotein electron transfer.

*E*_a_ further revealed the thermodynamic consequences of flavin binding. In the absence of flavins, *E*_a_ increased substantially in both Δ*omcA* and Δ*mtrC* biofilms due to the impaired clonal interprotein interactions (Figure 2E). Remarkably, the addition of FMN to Δ*omcA* or RF to Δ*mtrC* restored *E*_a_ to ∼0.3 eV, matching the value observed in WT biofilms with flavins (Figures 4D, S16 and S17). In contrast, the reciprocal flavin additions (RF to Δ*omcA* or FMN to Δ*mtrC*) failed to restore the conduction thermodynamics. Cholesterol addition significantly increased *E*_a_ for the flavin-mediated electron conduction, indicating that diffusion and collision govern *I*_cond_ at −150 mV as well (Figures 4D and S18). These observations suggest that specific flavin–OMC interactions thermodynamically enhance LDET by promoting interprotein affinity in a highly selective manner, which is distinct from nonspecific flavin binding affinity in the biofilm condition.

Conditions that facilitated favorable OMC–flavin interactions (WT + RF/FMN, Δ*omcA* + FMN, and Δ*mtrC* + RF) consistently produced highly reproducible *E*_a_ values with minimal variance (Figure 4D), while other conditions resulted in greater variability, likely reflecting lower-affinity, less stable protein–protein interactions. These differences are consistent with the notion that high-affinity interactions, such as those stabilized by structural complementarity, support robust electron transfer, while weaker interactions are more sensitive to fluctuations in diffusional and collisional dynamics.^28^ Altogether, these results support a model in which direct inter-OMC electron transfer underlies the LDET within the biofilm matrix.

To further test whether flavin binding alters the spatial alignment of heme centers within MtrC and OmcA, we performed CD spectroscopy on Δ*omcA* and Δ*mtrC* cell suspensions in the presence of flavins. Under reducing conditions, the CD spectra of MtrC with RF and OmcA with FMN were nearly identical to those obtained without flavins (Figure 4E,F), indicating that flavin binding does not disrupt the heme packing site, consistent with the location of the flavin binding site outside of domains containing the heme centers.^6,29^ These results support the conclusion that the addition of flavin did not alter the interheme interaction in OMCs, and the bound flavin blocked direct heme–heme electron flow.

### Effect of Temperature Sweeping on Heme Alignment in OMCs

Additionally, we confirmed that the temperature dependency of interheme coupling in OMCs is negligible using CD spectroscopy. Given that interprotein electron transfer is the rate-limiting step, the heme oxidation state is expected to be reduced more under in vivo conditions. Under reductive and oxidative conditions, the CD ellipticity of *S.*MR-1 changed by less than 6% across a temperature range of 36 to 11 °C (Figure S19A). A similar trend was observed in Δ*omcAll*, which represents the background CD signal for OMCs in the WT (Figure S19C–E). This minimal temperature dependence in CD signals indicates that heme alignment within the OMCs is not a primary determinant of the temperature dependency of *I*_cond_. These findings suggest that interheme electron coupling has a negligible impact on *E*_a_, further supporting the conclusion that interprotein interactions between OMCs on the cell surface dominate biofilm electron conduction.

## Discussion

While LDET through biofilms has been extensively studied at both microbial population and molecular levels, identifying the rate-limiting step has remained a significant challenge.^7,10,11^ Because the molecular structures of electron-mediating proteins have been resolved, most studies have focused on intraprotein electron transfer processes, often overlooking the critical role of interprotein electron transfer. In this study, we provide the first direct evidence that the diffusion and collision of the OMCs on the outer membrane mediate electron transfer through a rate-limiting interprotein interaction in biofilms by genetically deleting the OMCs and modulating membrane fluidity (Figures 2 and 3). This finding aligns with the crystal structure of the MtrCAB complex, where heme 10 receives electrons from MtrA.^6^ Additionally, lateral electron transfer pathways involving hemes 2 and 7 have been demonstrated in gene-engineered *E. coli*, where enhanced interheme coupling at these sites significantly improved lateral electron conduction.^16^ These insights provide a stronger foundation for understanding the cross-dagger structure of deca-heme cytochromes, which appear to be evolutionarily optimized for both lateral and vertical electron transfer against the outer membrane,^30^ facilitating biofilm development on electron acceptor surfaces.

The present study also demonstrates that the affinity between OmcA and MtrC facilitates LDET in biofilms, providing insight into mechanisms that regulate electron transfer pathways and kinetics on the outer membrane (Figure 2E). While the diffusion of OMCs on the membrane of *S.*MR-1 has been previously observed using quantum dot-based imaging techniques,^8^ stoichastic electron hopping among OMCs has been generally assumed. Our findings suggest that the affinity between OmcA and MtrC likely originates from structural compatibility near hemes 2 and 7. Given that interprotein interactions between structurally flexible MtrC and OmcA^6,16,31^ would involve conformational changes, validating and elucidating this interaction will require advanced protein docking analyses or cocrystallization studies of OmcA and MtrC.^5,32,33^ This novel insight could significantly advance our understanding of the dynamic alignment of heme groups within the unique deca-heme cytochrome complexes in vivo.^6,34,35^

Flavin binding to OMCs thermodynamically enhances electron exchange and increases the interprotein affinity between clonal protein pairs, such as OmcA and MtrC, interactions that are otherwise less favorable in the absence of flavins (Figure 2E). By altering the interprotein affinity, flavin binding improves the efficiency of electron exchange via diffusion and collision. In addition, these findings suggest that the presence of flavins enhances electron transfer within biofilms both qualitatively and quantitatively (Figures S16 and S17), underscoring the importance of optimizing protein interfaces. Given the natural accumulation of secreted flavins, such as RF and FMN,^36^ within *S.* MR-1 biofilms, the organism appears to utilize a low-barrier, high-stability electron conduction mechanism for biofilm maturation.

Because upon adding FMN to the WT biofilm, *I*_cond_ showed a reduction of over 70% (Figure 4B inset), interprotein electron transfer through the OMCs is the primary mechanism for electron conduction in the WT biofilm. However, it is notable that *I*_cond_ did not drop to zero in the Δ*omcAll* strain. This residual electron conductivity suggests the presence of an alternative electron transfer pathway, particularly at +120 mV, which operates independently of the OMC-mediated heme redox centers (Figure S3). One possibility is the involvement of a soluble redox-active molecule biosynthesized by Δ*omcAll*, consistent with the disordered temperature dependence observed—like that seen in cell-free riboflavin systems. Given the relatively positive redox potential, menaquinone rather than riboflavin is a more likely candidate for this alternative mediator. Such soluble redox species may associate with extracellular DNA, as has been reported for phenazines in *Pseudomonas* biofilms.^37^ The presence of such a pathway could also help explain the nonlinear and complex behavior of peak currents when varying flavin concentration (Figure 4A,B), reflecting overlapping contributions from both membrane-bound and diffusible electron carriers.

Our findings open new avenues for leveraging synthetic biology to integrate flavin biosynthetic pathways, enabling the creation of highly conductive biofilms for biocatalysis. Furthermore, targeted genetic modifications to MtrC or OmcA, coupled with the identification of more effective bound cofactors than flavins, could further enhance electron transfer rates in biofilm systems by optimizing interprotein electron transfer.^21,38^ The significance of protein interfaces in mediating efficient electron transfer is also evident in other LDET systems, such as the protein nanowires in *Geobacter* species,^34^ suggesting a potentially universal mechanism for LDET kinetics in biofilms.

## Conclusion

While significant progress has been made in elucidating the roles of MtrC and OmcA in EET, our study challenges the long-standing assumption that intraprotein electron transfer within the OMCs is the rate-limiting step for biofilm electron conduction. Instead, we identified key heme centers facilitating dominant inter-OMC electron transfer pathways, providing a new perspective on the mechanisms governing biofilm conductivity. These findings enhance our understanding of biofilm electron conduction and pave the way for engineering highly conductive biofilms through targeted protein interface modifications. Such advancements hold great promise for the development of next-generation bioelectric devices, poised to drive future innovations.

## Supplementary Material



## Notes

Experimental details, computational methodology, additional characterization, and controls (PDF)
