# Supplementary material for: Cell-Surface Inter-Cytochrome Electron Transfer Limits Biofilm Electron Conduction Kinetics in Shewanella oneidensis
Source: J Am Chem Soc. 2025 Dec 15;147(51):46932–40. doi: 10.1021/jacs.5c10357 (PMC12750994; doi:10.1021/jacs.5c10357)
Supplement: Supplementary file 1 [file ja5c10357_si_001.pdf]

Supporting Information

Cell-surface Inter-Cytochrome Electron Transfer Limits Biofilm Electron Conduction Kinetics in *Shewanella oneidensis*

Xinxin Wen[a,c,§], Xizi Long[a,b,§,*], Wenyuan Huang[a,c], Masahiro Kuramochi[d,e], Akihiro Okamoto[a,c,f,g,*]

[a] *Research Center for Macromolecules and Biomaterials, National Institute for Materials Science, Tsukuba, Ibaraki 305-0044, Japan*

[b] *Key Laboratory of Typical Environmental Pollution and Health Hazards of Hunan Province, School of Public Health, Hengyang Medical School, University of South China, Hengyang 421001, China*

[c] *Graduate School of Chemical Sciences and Engineering, Hokkaido University, North 13 West 8, Kitaku, Sapporo, Hokkaido 060-8628, Japan*

[d] *Graduate School of Science and Engineering, Ibaraki University, Hitachi, Ibaraki 316-8511, Japan*

[e] *Graduate School of Frontier Sciences, The University of Tokyo, Kashiwa, Chiba 277-8561, Japan*

[f] *Graduate School of Science and Technology, University of Tsukuba, 1-1-1 Tennodai, Tsukuba, 305-8577, Japan*

[g] *Research Center for Autonomous Systems Materialogy, Institute of Integrated Research, Institute of Science Tokyo (Science Tokyo), 4259 Nagatsuta-cho, Midori-ku, Yokohama, Kanagawa 226-8503, Japan*

[§] *X.X.W. and X.Z.L. contributed equally to this work.*

Contents Summary:

Figures S1-S19 (page 2-13)

References (page 14)

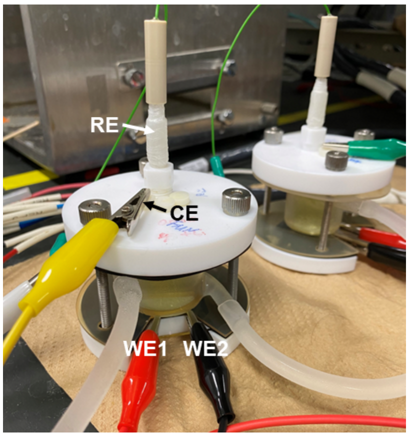

Figure S1. Photograph of the experimental four-electrode setup. The reference electrode (RE) is Ag/AgCl (saturated KCl). Counter electrode (CE) is a Pt wire. Interdigitated working electrodes 1 (WE1) and 2 (WE2) are indium tin-doped oxide (ITO) coated on a glass substrate.

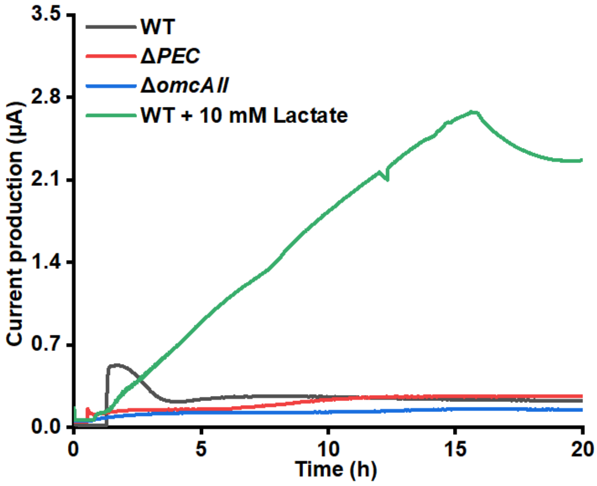

Figure S2. Single-potential amperogram of *Shewanella oneidensis* MR-1 (*S.*MR-1) wild-type (WT) and mutant strains, Δ*PEC* and Δ*omcAll*. The time-dependent current profiles were measured (30 °C, 0.4 V vs SHE) after washing biofilm-attached WE1 and WE2 with defined medium to remove the electron donor, sodium lactate. The green-colored profile represents the current profile measured in the presence of 10 mM lactate.

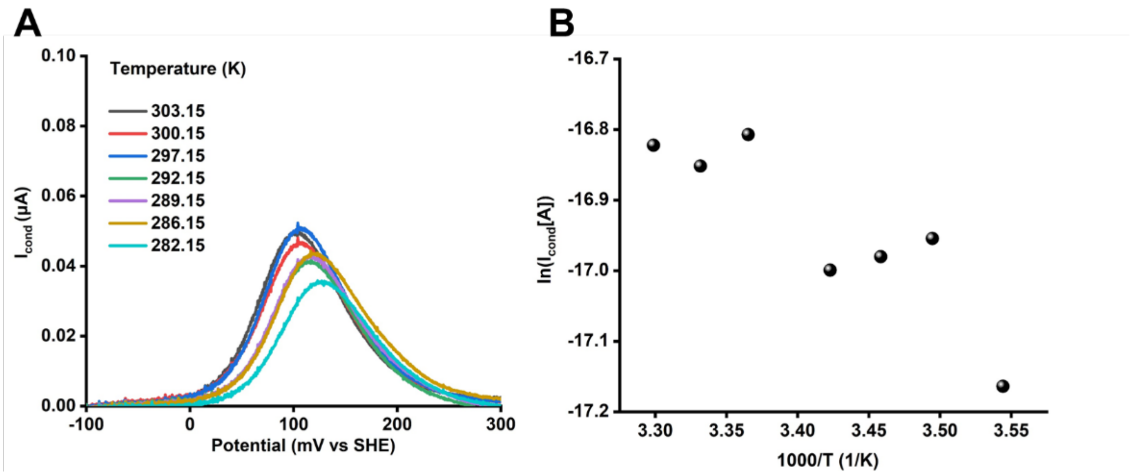

Figure S3. Temperature dependence of conductive current (I_{cond}) in Δ*omcAll*. (A) Representative I_{cond} profiles at various temperatures as a function of gate potential after 24 hours of incubation on interdigitated ITO electrodes at 0.4 V vs SHE. (B) Plots for the exponential factor of I_{cond} against the reciprocal of absolute temperature showed no correlation, indicating the temperature dependence of I_{cond} is not solely governed by a conventional thermally activated process.

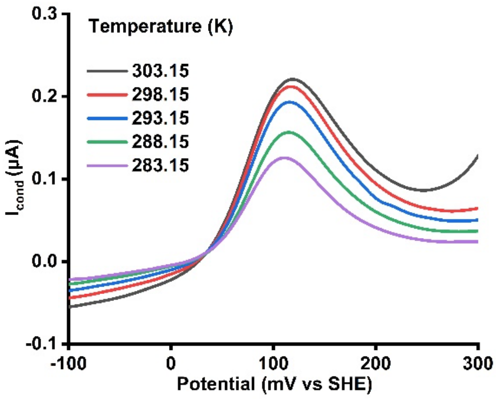

Figure S4. Temperature dependence of I_{cond} in Δ*PEC*. Representative I_{cond} profiles at various temperatures as a function of gate potential in Δ*PEC* after 24 hours of incubation on interdigitated ITO electrodes at 0.4 V vs SHE.

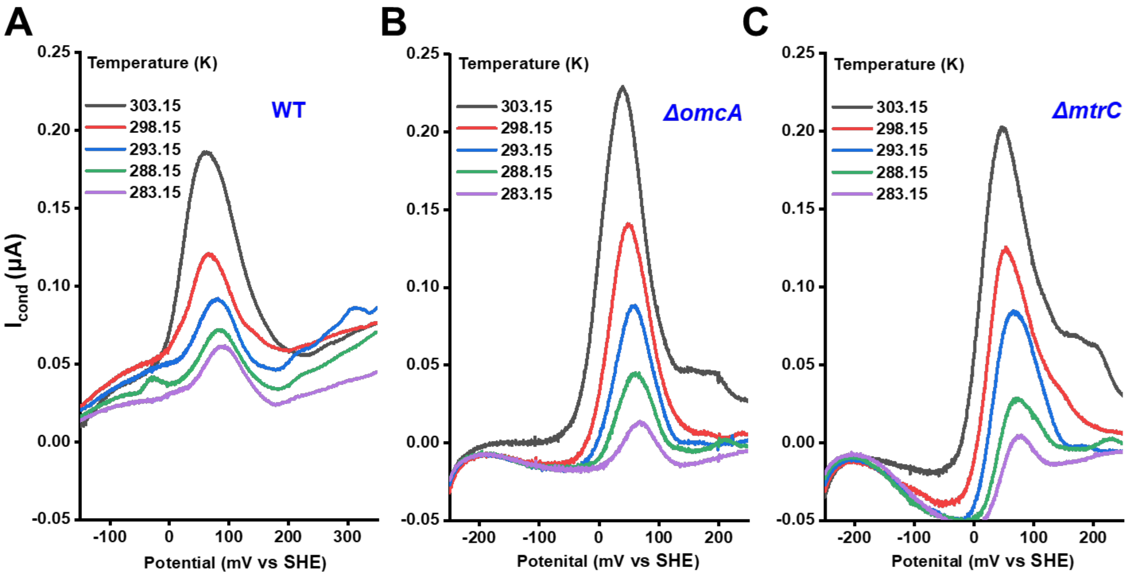

Figure S5. Temperature dependence of I_{cond} in (A) WT, (B) Δ*omcA* and (C) Δ*mtrC*. Representative I_{cond} profiles at various temperatures as a function of gate potential after 24 hours of incubation on interdigitated ITO electrodes at 0.4 V vs SHE in the presence of 10 mM sodium lactate.

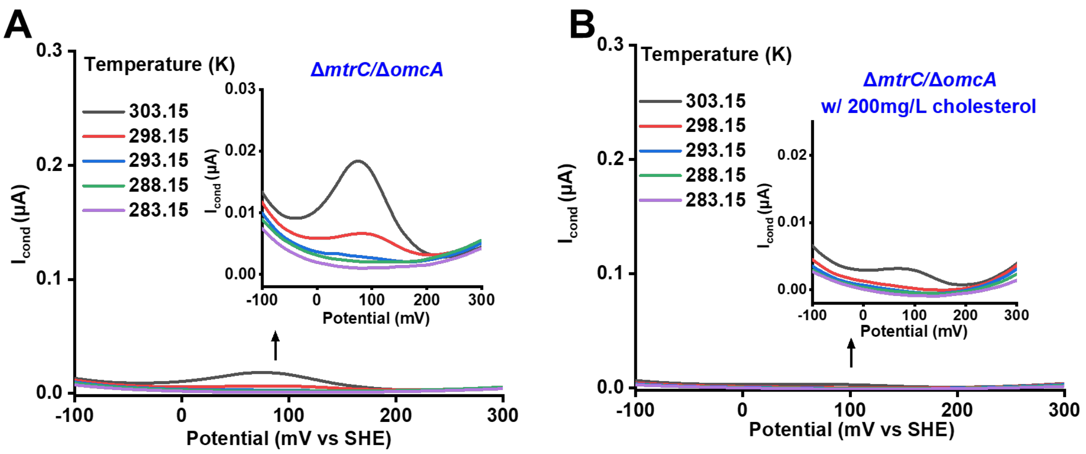

Figure S6. Temperature dependence of I_{cond} in Δ*mtrC*/Δ*omcA* without (A) and with (B) preculturing with 200 mg/L cholesterol. Representative I_{cond} profiles at various temperatures as a function of gate potential after 24 hours of incubation on interdigitated ITO electrodes at 0.4 V vs SHE.

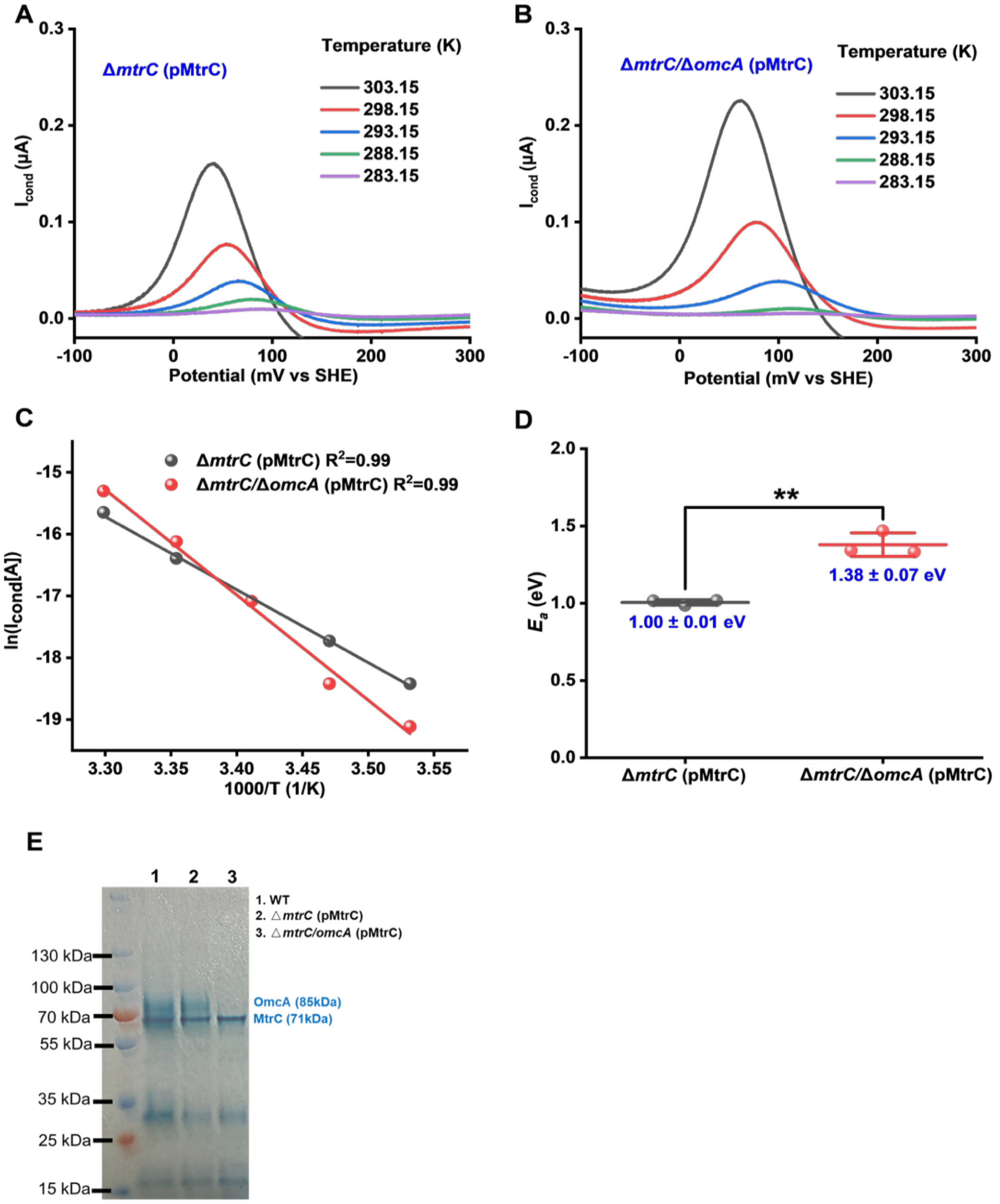

Figure S7. Temperature dependence of I_{cond} in complemented strains (A) Δ*mtrC* (pMtrC) and (B) Δ*mtrC*/Δ*omcA* (pMtrC). Representative I_{cond} profiles at various temperatures as a function of gate potential after 24 hours of incubation on interdigitated ITO electrodes at 0.4 V vs SHE. (C) Plots for the exponential factor of I_{cond} against the reciprocal of absolute temperature. (D) E_a estimated from the temperature dependence of I_{cond} for each strain. Data are presented as mean ± s.d. (n = 3). **p < 0.01. (E) Protein profiles of WT, Δ*mtrC* (pMtrC) and Δ*mtrC*/Δ*omcA* (pMtrC) cells after growth in LB medium before the electrochemical assays were stained with heme-reactive 3,3′,5,5′-tetramethylbenzidine-H_2O_2.

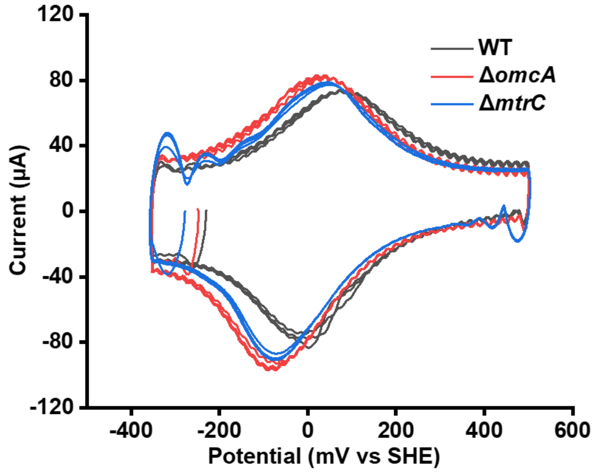

Figure S8. Cyclic voltammograms of WT, Δ*omcA* and Δ*mtrC* recorded at the scan rate of 1000 mV s^{-1} after 24 hours of incubation on the interdigitated ITO electrodes at 0.4 V vs SHE.

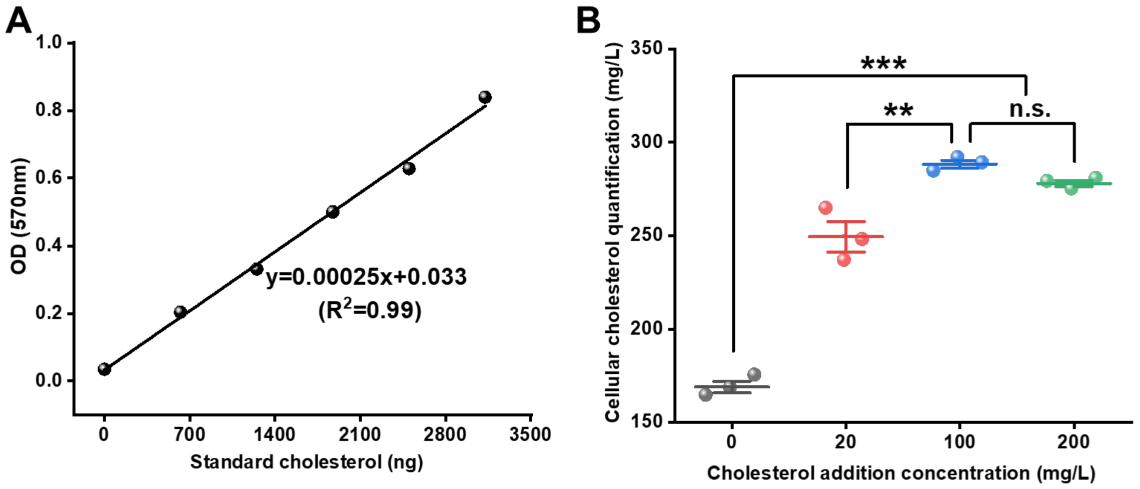

Figure S9. Standard calibration curve for cholesterol (A), and the quantification of intracellular cholesterol levels after electrochemical measurement for estimating E_a (B). Data are presented as mean ± s.d. (n = 3). n.s., not significant; **p < 0.01; ***p < 0.001.

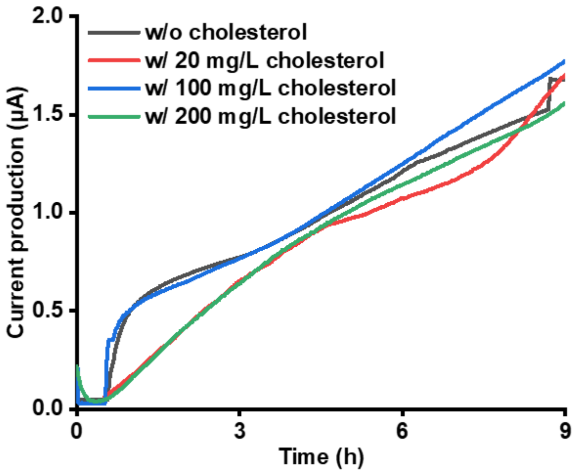

Figure S10. Single-potential amperograms of *S*.MR-1 cells treated with varying concentrations of cholesterol, recorded at 30 °C and 0.4 V vs SHE in the presence of 10 mM sodium lactate.

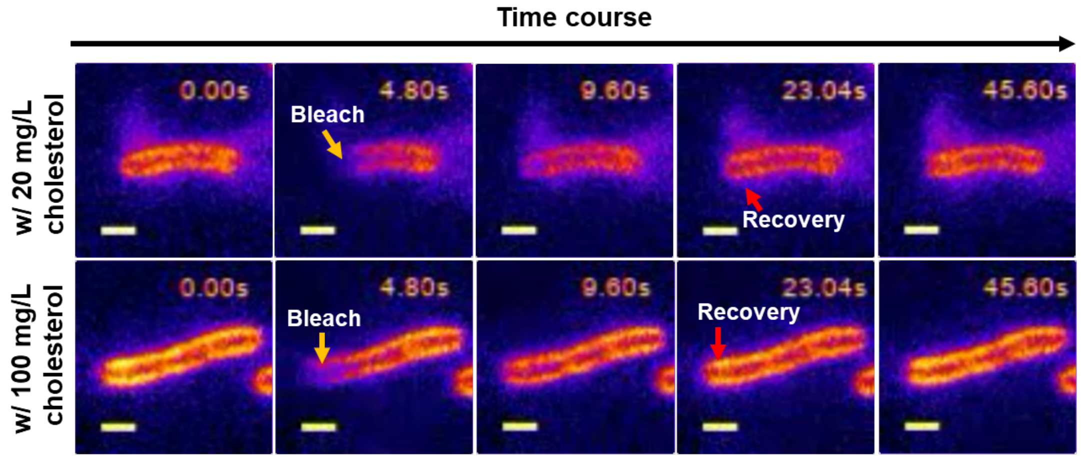

Figure S11. Time-course images of *S*.MR-1 cells stained with FM4-64FX for detecting fluorescence recovery after photobleaching at the cholesterol concentrations of 20 and 100 mg/L.

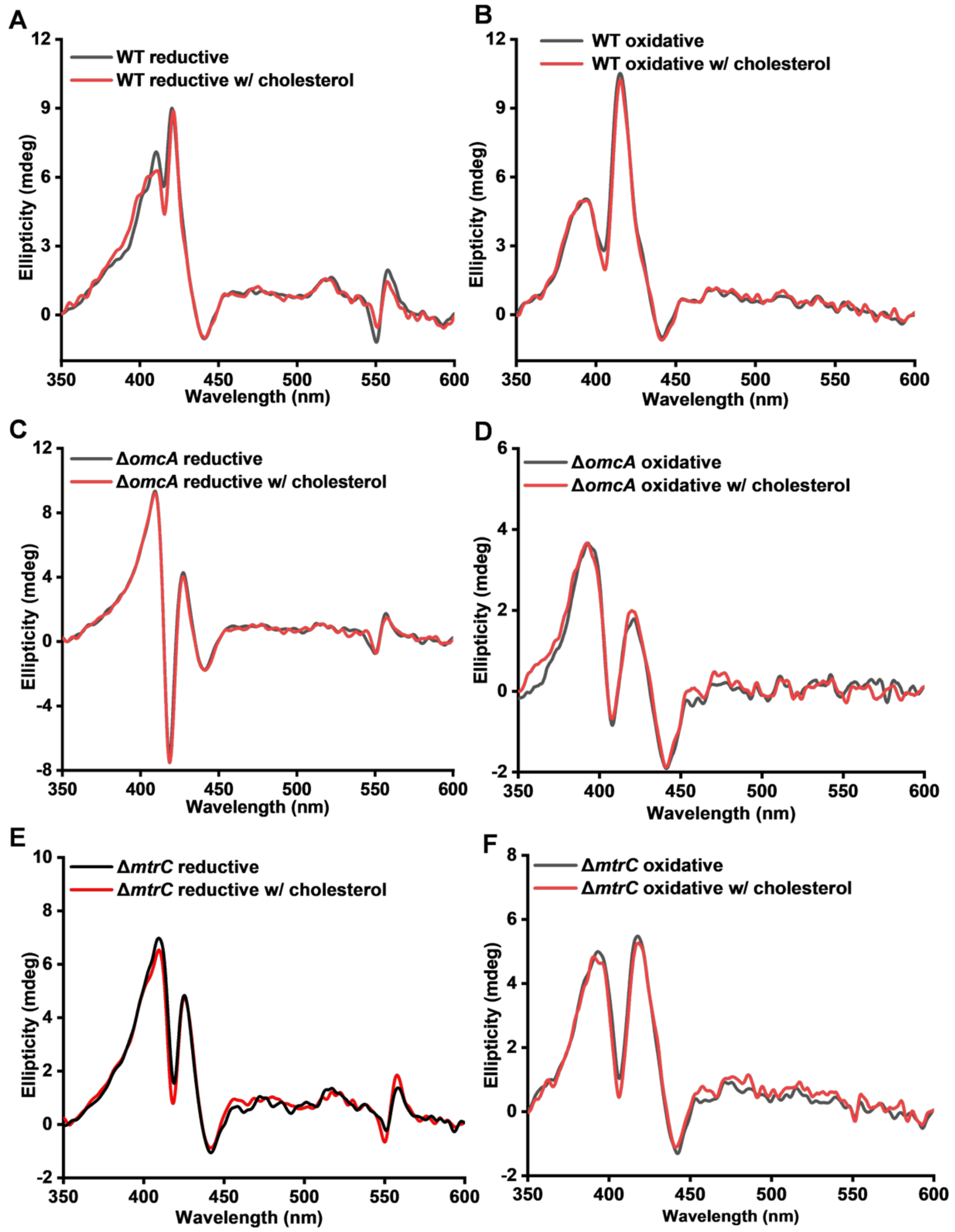

Figure S12. Circular dichroism spectra of *S*.MR-1 WT, Δ*omcA* and Δ*mtrC* under reducing (A, C, E) and oxidizing (B, D, F) conditions, with and without cholesterol preculturing.

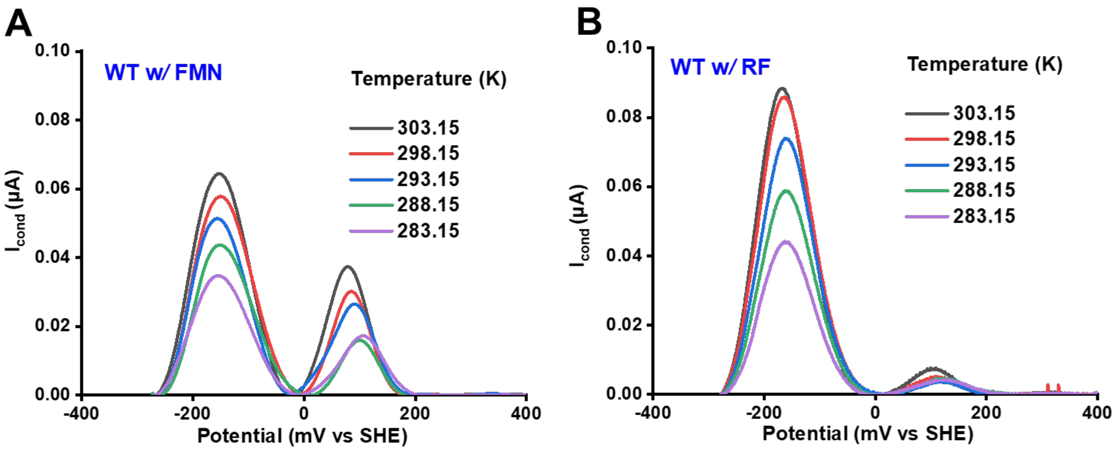

Figure S13. Representative temperature dependence of I_{cond} versus the gate potential in the presence of 10 μM RF (A) and FMN (B) in WT. Notes: for RF and FMN-mediated conductions, the baseline subtraction was performed using Origin's Peak Analyzer tool with the "User Defined" mode. Points within ±100 mV of the conduction peak potential at each temperature were selected to isolate conduction currents assignable to the flavin or heme-mediated mechanism. This method minimizes contributions from background noise, enabling precise quantification of the bound flavin. The same data analysis method was applied to the following figures (Figure S16-S18).

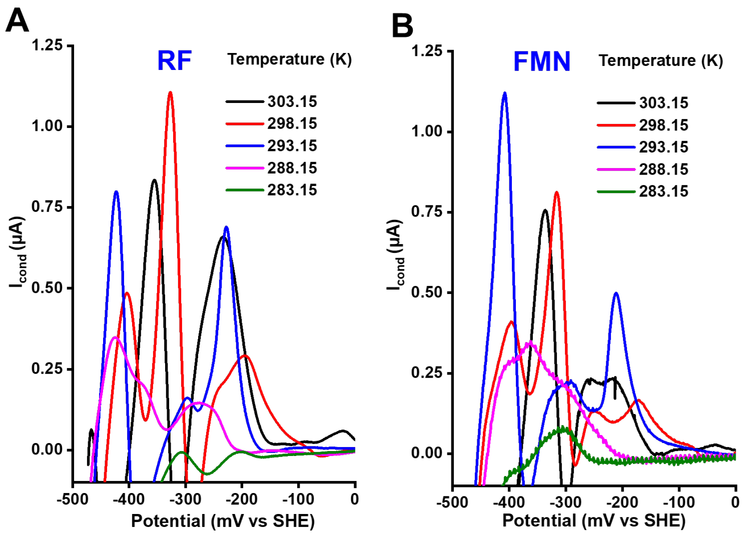

Figure S14. I_{cond} profiles versus the gate potential in the absence of bacterial cells, at various temperatures with 10 μM RF (A) and 10 μM FMN (B).

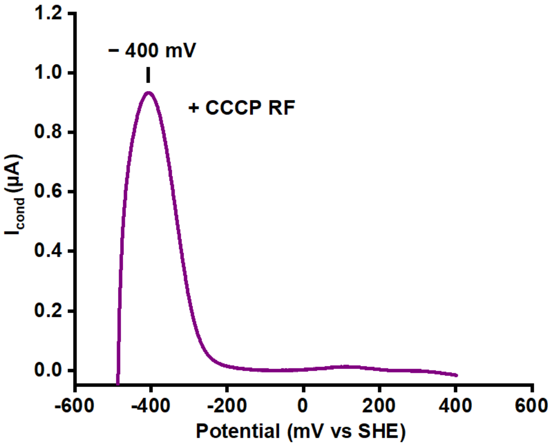

Figure S15. An I_{cond} profile versus the gate potential in the presence of *S.*MR-1 biofilm and 10 µM riboflavin after adding 50 µM carbonyl cyanide m-chlorophenylhydrazone (CCCP) at 30 ˚C.

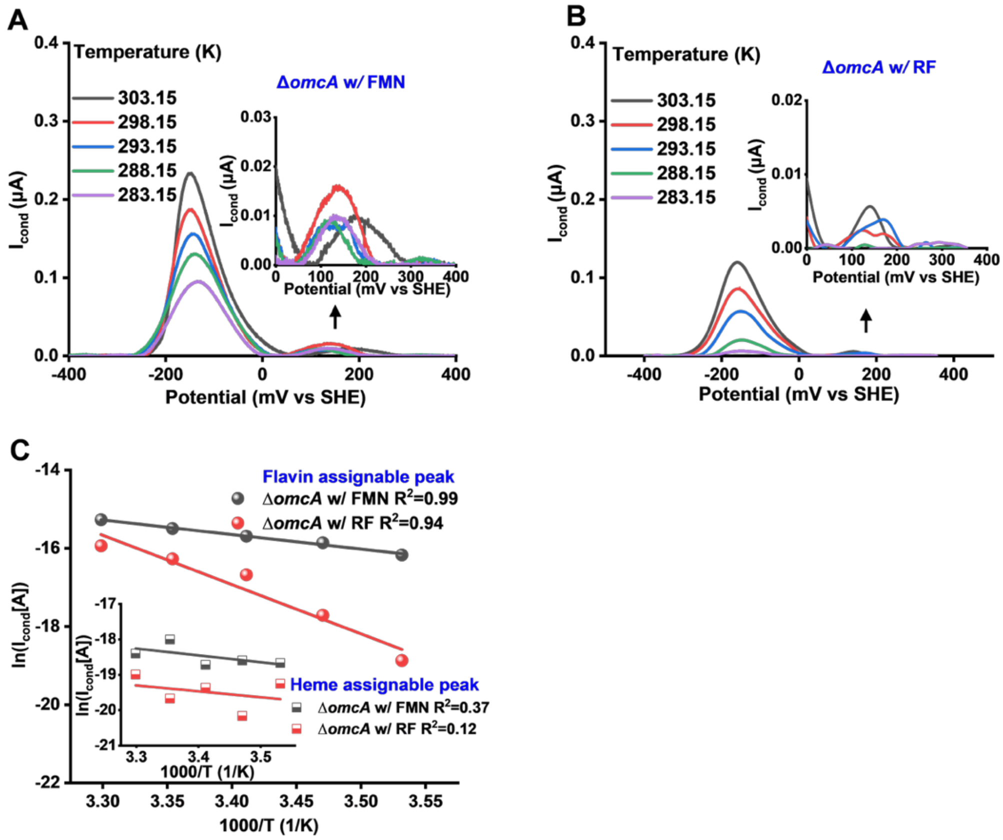

Figure S16. Representative temperature dependence of I_{cond} versus the gate in the presence of 10 μM FMN (A) and RF (B) with Δ*omcA*. (C) Representative Arrhenius-style plots of Δ*omcA* in the presence of 10 μM RF and FMN.

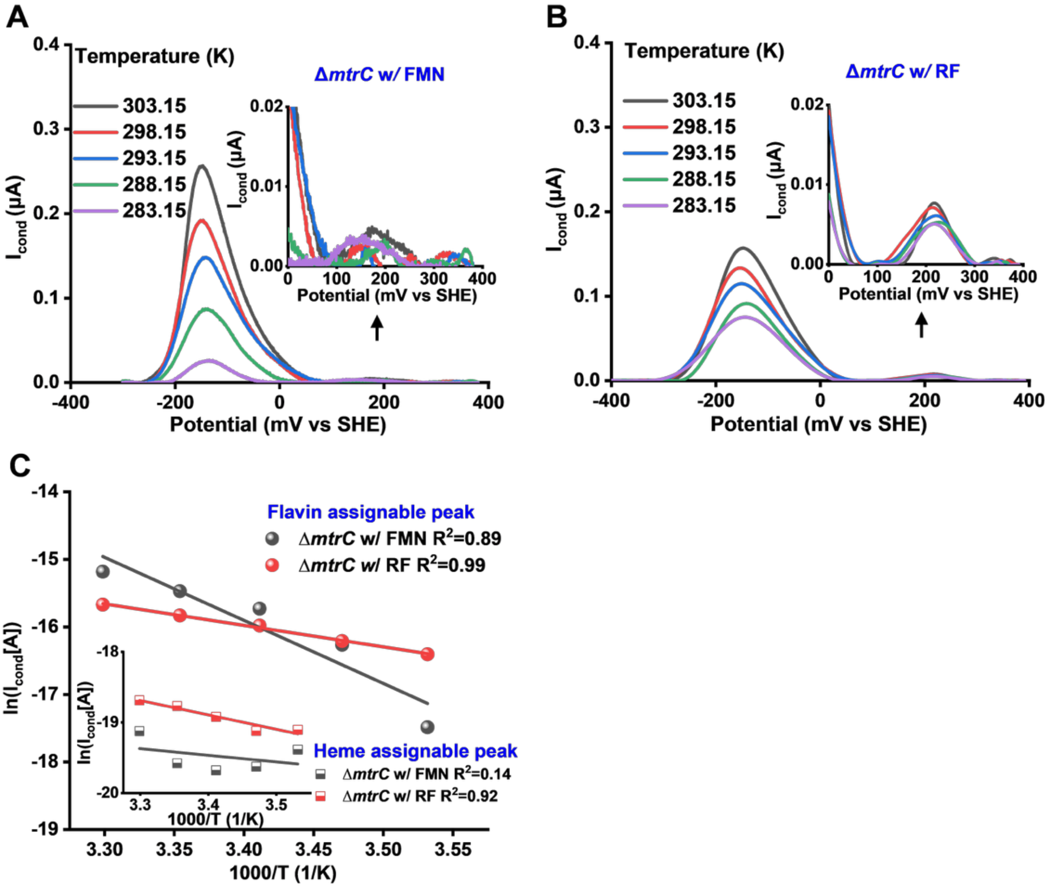

Figure S17. Representative temperature dependence of I_{cond} versus the gate potential in the presence of 10 μM FMN (A) and RF (B) with Δ*mtrC*. (C) Representative Arrhenius-style plots of Δ*mtrC* in the presence of 10 μM RF and FMN.

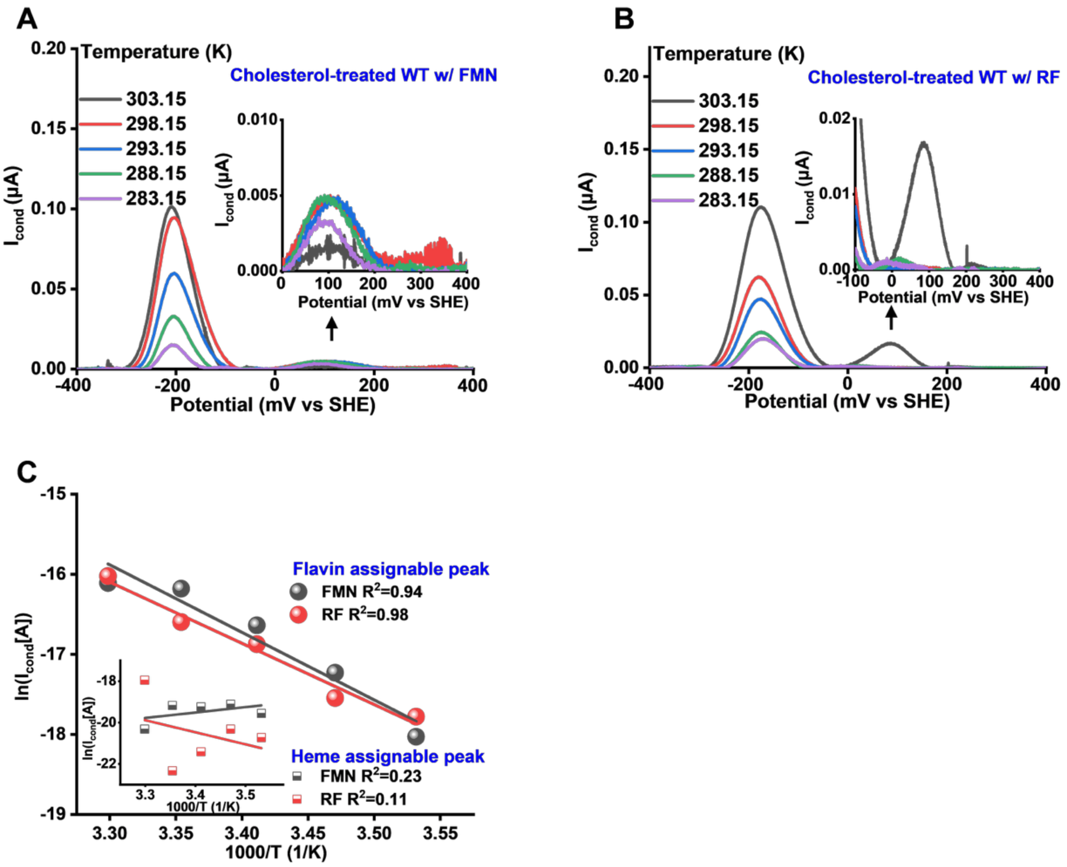

Figure S18. Representative temperature dependence of I_{cond} versus the gate potential in the presence of 10 µM FMN (A) and RF (B) after cholesterol preincubation (200 mg/L) with WT. (C) Representative Arrhenius-style plots in the presence of 10 µM FMN and RF.

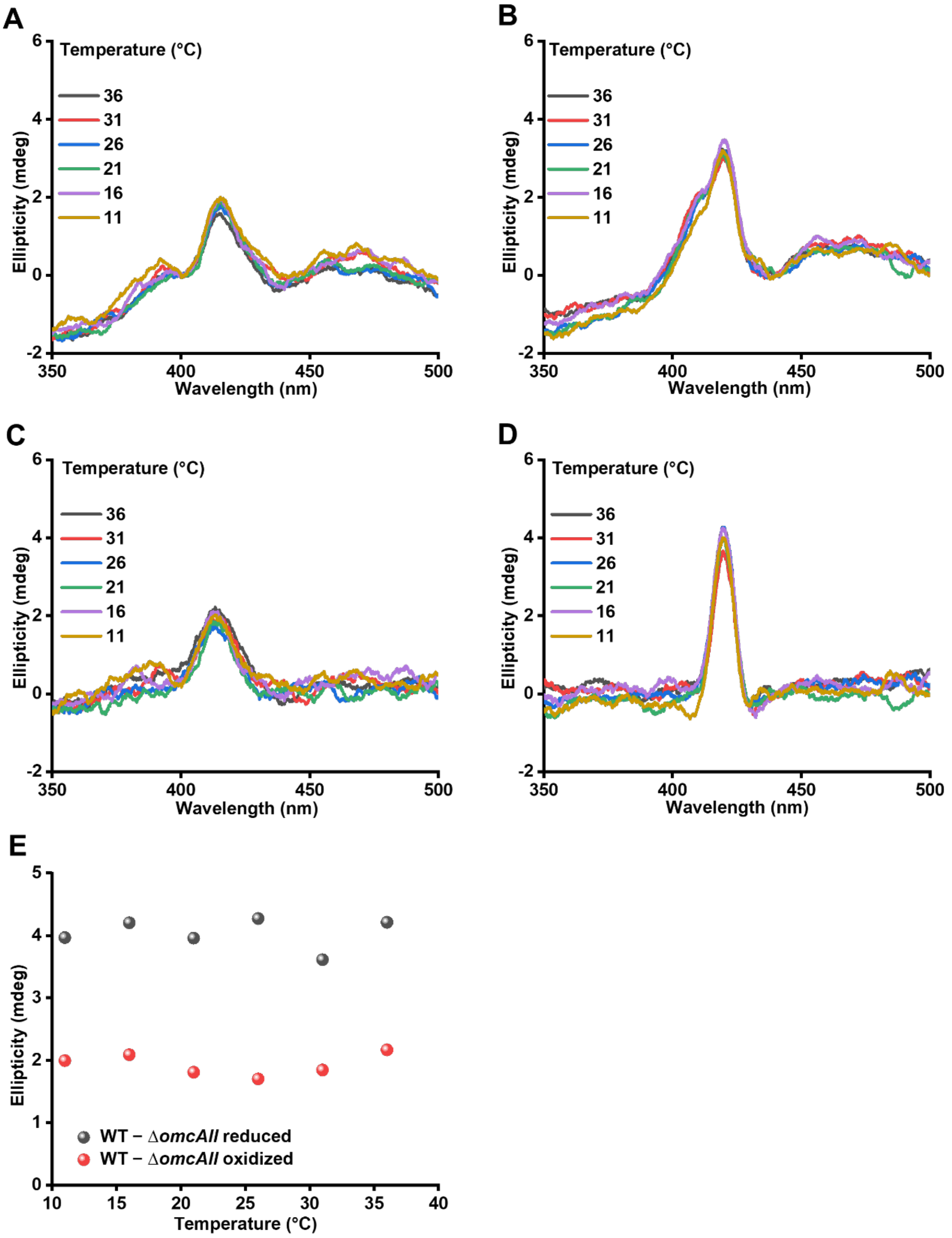

Figure S19. Circular dichroism (CD) spectra of *S*.MR-1 were collected at multiple temperatures under (A) oxidative and (B) reductive conditions. Whole-cell CD difference spectra were obtained by subtracting the spectra of Δ*omcAll* from those of the wild type (WT) under (C) oxidative and (D) reductive conditions. (E) The peak ellipticity values for the CD difference spectra of OMCs are presented for both oxidative and reductive conditions over a range of temperatures.

Reference:

1. Eaton, L.; Erdos, G.; Vreeland, N.; Ingram, L. J., Failure of *Escherichia coli* to alter its fatty acid composition in response to cholesterol-induced changes in membrane fluidity. *J. Bacteriol.* **1981,** *146* (3), 1151-1153.
2. Tokunou, Y.; Okamoto, A., Geometrical changes in the hemes of bacterial surface c-type cytochromes reveal flexibility in their binding affinity with minerals. *Langmuir* **2018,** *35* (23), 7529-7537.
3. Tokunou, Y.; Chinotaikul, P.; Hattori, S.; Clarke, T. A.; Shi, L.; Hashimoto, K.; Ishii, K.; Okamoto, A., Whole-cell circular dichroism difference spectroscopy reveals an in vivo-specific deca-heme conformation in bacterial surface cytochromes. *Chemical Communications* **2018,** *54* (99), 13933-13936.
4. Long, X.; Okamoto, A., Outer membrane compositions enhance the rate of extracellular electron transport via cell-surface MtrC protein in *Shewanella oneidensis* MR-1. *Bioresource Technology* **2021,** *320*, 124290.
5. Xu, S.; Barrozo, A.; Tender, L. M.; Krylov, A. I.; El-Naggar, M. Y., Multiheme cytochrome mediated redox conduction through *Shewanella oneidensis* MR-1 Cells. *Journal of the American Chemical Society* **2018,** *140* (32), 10085-10089.
6. Long, X.; Tokunou, Y.; Okamoto, A., Mechano-control of extracellular electron transport rate via modification of inter-heme coupling in bacterial surface cytochrome. *Environmental Science & Technology* **2023,** *57* (19), 7421–7430.
